# The Effects of Assets and Debt on the Depression Trajectory of Low-Income Older Adults in South Korea

**DOI:** 10.1093/geroni/igab046.1939

**Published:** 2021-12-17

**Authors:** Jihee Woo, Hyojin Choi

**Affiliations:** 1 University of Pittsburgh, Pittsburgh, Pennsylvania, United States; 2 Sungkyunkwan University, Seoul, Seoul-t'ukpyolsi, Republic of Korea

## Abstract

Evidence suggests that assets and debt are among the most significant factors affecting older adults’ mental health. This study focuses specifically on South Korea, where the poverty rate of older adults is the highest among all OECD countries. Given that low-income older adults with fewer assets and more debt may be at greater risk for depression, we investigated how assets and debt affected the depression trajectory of low-income older adults in South Korea. We used the six most recent waves of data from the Korean Welfare Panel Study (2014-2019) to estimate the longitudinal effects of assets and debt on depression in low-income older adults. Our sample was restricted to low-income Korean heads of household aged 55 and above (N=2,832). Using latent growth curve modeling, the unconditional model revealed decreasing trends in depression over time, while the conditional model, controlling for sociodemographic variables (i.e., age, gender, education, general health, marital status, employment status, income), suggested that assets and debt had contrasting impacts on depression. Specifically, although it did not impact the depression trajectory, debt did have a positive impact on depression at baseline. Most notably, assets negatively affected both depression at baseline (B=-1.911, SE=0.284, p<.001) and its trajectory (B=-0.235, SE=0.081, p<.01). These findings highlight the importance of holding assets over time as a protective factor against depression and thus the need for interventions such as savings programs and financial education for low-income older adults.

